# Unsupervised clustering analysis of comprehensive health status and its influencing factors on women of childbearing age: a cross-sectional study from a province in central China

**DOI:** 10.1186/s12889-023-17096-3

**Published:** 2023-11-09

**Authors:** Lu He, Si-Tian Li, Meng-Xia Qin, Yan Yan, Yuan-Yuan La, Xi Cao, Yu-Tong Cai, Yu-Xiao Wang, Jie Liu, Da-Hong Wu, Qilong Feng

**Affiliations:** 1 Department of Social Medicine, School of Public Health, Shanxi Medical University, Taiyuan, Shanxi 030001 People’s Republic of China; 2 Ministry of Education, Key Laboratory of Coal Environmental Pathopoiesis and Control at Shanxi Medial University, Taiyuan, Shanxi 030001 People’s Republic of China; 3 Department of Contingency Management, Shanxi Provincial People’s Hospital, Taiyuan, Shanxi 030001 People’s Republic of China; 4 School of Social Development and Public Policy, Beijing Normal University, Beijing, 100000 People’s Republic of China; 5 Department of Health Economics, School of Management, Shanxi Medical University, Taiyuan, Shanxi 030001 People’s Republic of China; 6 Department of Physiology, Key Laboratory of Cellular Physiology, Ministry of Education, Shanxi Medical University, Taiyuan, Shanxi 030001 People’s Republic of China

**Keywords:** Women’s health, Women of childbearing age, Cluster analysis, Correlation, Multiple regression

## Abstract

**Background:**

Most previous studies on women of childbearing age have focused on reproductive health and fertility intentions, and evidence regarding the comprehensive health status of women of childbearing age is limited. This study aimed to comprehensively examine the health status of women of childbearing age through a multi-method and multi-indicator evaluation, analyze the factors that influence their overall health, and provide sound recommendations for the improvement and promotion of healthy behaviors.

**Methods:**

Data on women of childbearing age living in Shanxi Province were collected between September 2021 and January 2022 through online and offline surveys. The k-means algorithm was used to assess health-related patterns in women, and multivariate nonconditional logistic regression was used to assess the influencing factors of women’s overall health.

**Results:**

In total, 1,258 of 2,925 (43%) participants were classified as having a good health status in all five domains of the three health dimensions: quality of life, mental health, and illness. Multivariate logistic regression showed that education level, gynecological examination status, health status of family members, access to medical treatment, age, cooking preferences, diet, social support, hand washing habits, attitude toward breast cancer prevention, and awareness of reproductive health were significantly associated with different health patterns.

**Conclusions:**

The comprehensive health status of women of childbearing age in Shanxi Province is generally good; however, a large proportion of women with deficiencies in some dimensions remains. Since lifestyle greatly impacts women’s health, health education on lifestyle and health-related issues should be strengthened.

**Supplementary Information:**

The online version contains supplementary material available at 10.1186/s12889-023-17096-3.

## Background

The health status of childbearing-aged women is an important issue that affects not only the survival and development of the female population but also the health of family members, especially the next generation. According to the World Health Organization, women have a longer life expectancy than men in most countries. However, several health and social factors result in a lower quality of life in women [1]. Women bear the burden of disease disproportionately and face premature death due to sex-based inequities. Significant differences exist between men and women regarding access to basic healthcare services, nutrition, and educational opportunities [2]. A recent report showed that globally, the levels of stress, anxiety, worry, sadness, and anger among women were at a ten-year high [3]. The question of how to improve fertility rates and the health of women of childbearing age has become one of the most discussed population-related issues today.

Prolonged exposure to work-related stress and excessive household chores can cause various physical and mental health challenges [4]. These may manifest as fatigue, disrupted sleep patterns, headaches, muscular tension, and physical strain. Cumulatively, these factors can contribute to the development of chronic conditions, including musculoskeletal disorders, cardiovascular complications, and compromised immune system functionality. Moreover, stress, extended work hours, and inadequate rest periods can contribute to heightened levels of anxiety, depression, and burnout [5]. Some studies have indicated that women spend a disproportionate amount of time doing three-quarters of the world’s unpaid work (including personal care and housework) [6]. Moreover, unpaid domestic and caretaking work is associated with a greater mental health burden and negative effects on the quality of life of women [7]. Further, the coronavirus disease (COVID-19) pandemic has exacerbated the economic and health stress faced by women due to the impact of layoffs, changes in the work environment, pandemic-related unemployment due to access (or lack thereof) to healthcare [8], and the intensity of unpaid work performed by women. Hologic, a medical technology company, partnered with Gallup to launch a global survey of women aged ≥ 15 years in 122 countries and territories to assess how well women’s health needs were being met. In 2021, the Hologic Global Women’s Health Index score was 53 out of 100, which was 1 point lower than that in 2020 [3]. Ginsburg et al. reported that the current disease burden of breast and cervical cancer remains high among women worldwide and that efforts are urgently needed to address the threat of malignancies to women’s health [9].

The health status of women of childbearing age is closely related to their level of fertility. The various aspects of fertility, encompassing pregnancy, infertility, and miscarriage, exert notable influences on a woman’s physical and mental health. Hormonal fluctuations associated with these reproductive events can impact mood and overall well-being. Moreover, women in this age group exhibit higher incidence rates of certain diseases, such as breast cancer and osteoporosis, and confront distinct health challenges pertaining to reproductive health, encompassing pregnancy-related complications and maternal mortality [10]. Over the past few years, fertility rates have declined in both high- and low-income countries [11, 12], and the problem of aging has become a serious burden. China is actively encouraging “two children” and “three children” policies in an effort to reverse the persistently low fertility rate [13]. Following the implementation of fertility policy adjustments, there has been a notable rise in the proportion of advanced maternal age and multiparous women, subsequently amplifying the healthcare requirements of women in their childbearing years. To cater to these escalating healthcare demands, a range of policies have been introduced, specifically targeting the enhancement of primary maternal and child health services [14]. Consequently, understanding the factors that influence the health status of women in this age group becomes increasingly crucial. By gaining insight into the determinants that impact women’s health, appropriate measures can be undertaken to effectively address and support women’s well-being, thereby mitigating health disparities.

Women’s health affects not only the survival and development of the female population itself, but also the health of family members, especially the next generation. A cohort study in the United States showed a healthy lifestyle (normal weight, healthy eating habits, adherence to physical activity, non-smoking, and moderate alcohol consumption) of mothers before pregnancy had a positive impact on the health of their offspring [15]. However, most of the studies on women of childbearing age have focused on reproductive health and fertility intentions.

In previous research on the health status of women of childbearing age, assessments have commonly relied on established scales or single indicators for health status measurement and evaluation. However, health encompasses multiple dimensions, including physical, mental, and social well-being. Relying solely on a single measure may fail to capture the entirety of health and neglect other crucial aspects. This study used a multidimensional clustering approach for a comprehensive evaluation of health status in women of childbearing age to integrate multiple perspectives. By using an unsupervised clustering method to analyze a mixed dataset of women aged 15 to 49 years in Shanxi Province, this study aimed to identify potential concentration trends in value distributions across various health self-assessment dimensions, mental health dimensions, recent and long-term illness dimensions, and regression models.

Clustering analysis as a method of machine learning is a way to illustrate potential concentration trends in value distributions, especially for users for whom there is no available distribution information and for whom classification by traditional categories was difficult [16]. For a given sample set, the k-means algorithm divides the sample set into k clusters according to the size of the distance between samples and keeps the points within the clusters as closely related as possible by successive iterations while making the distance between clusters as large as possible [17]. This unsupervised machine learning clustering method has been used to analyze biological data for various purposes, such as stratifying clinical patients for more appropriate treatment [18], revealing predictive patterns of disease and assessing survival rates [19], and predicting clinical outcomes for early and aggressive intervention [20]. However, there are few studies on the application of the algorithm on comprehensive health conditions, especially in women of childbearing age.

A more comprehensive picture of the health status of women of childbearing age and the factors that influence it will in turn inform strategies to promote healthy lifestyles in this group, improve the overall well-being of women, and indirectly impact the health of the next generation significantly.

## Methods

### Study population

Data were collected from 2,925 women of childbearing age living in Shanxi Province through a non-probabilistic combination of online and offline surveys conducted between September 2021 and January 2022. Participants had to be female, 15–49 years of age [21], Chinese speaking, and mentally sound; had to reside in Shanxi Province; had to have good cognitive and communication skills; and had to voluntarily participate in the survey. The survey was designed by members of the research team. The questionnaire (see Supplementary materials) comprised 135 questions on sociodemographic information, lifestyle, hygiene habits, social support, mental health status, and knowledge, attitudes, and behaviors related to gynecological diseases. The survey required 25 min for completion. Data did not include identifying information and were only accessed and analyzed by members of the research team. A total of 3,628 questionnaires were distributed, and 3,460 valid questionnaires were returned, resulting in a 95.27% return rate.

### Measurements

#### Investigated variables

Comprehensive health status was the dependent variable and primary outcome; it was measured according to self-rated health, two-week illness, and the prevalence of chronic disease, depression, or anxiety. Self-rated health status was measured using the Short-Form Health Survey 12 (SF-12). The SF-12 contains 12 questions and the following 8 scales: physical functioning, role functioning-physical, body pain, general health, vitality, social functioning, role functioning-emotional, and mental health. Each dimension is scored out of 100, with a higher score indicating better health [22]. Participants were asked whether they had any of the 16 chronic diseases mentioned in the questionnaire. Two-week illness status was utilized to investigate whether the participants were unwell in the past two weeks prior to the survey and how they were treated. Depression was measured using the Center for Epidemiologic Studies Depression Scale and categorized into three categories according to the total score: no, possible, or definite depressive symptoms. The degree of anxiety was measured using the seven-item Generalized Anxiety Disorder scale and divided into five categories according to the total score as follows: no (< 5 points), mild (< 10 points), moderate (< 14 points), moderate to severe (< 19 points), and severe (≥ 19 points) anxiety. The health status of women of childbearing age was classified by the k-means unsupervised clustering method by grouping the women with similar health statuses into the same subset.

#### Explained variables

The survey assessed numerous sociodemographic variables and lifestyle behaviors, including age, household registration (city or rural), height, weight, income, education, marital status, and type of medical insurance. Lifestyle behaviors included smoking, alcohol consumption, daily water intake, sleep, physical activity, occupational stress, and dietary habits. Hygiene habits were also assessed and included hand washing, bathing, sharing daily necessities, cleaning private parts, and gynecological examination. In addition, participant knowledge and behaviors related to gynecological diseases (breast cancer, cervical cancer) and reproductive health were assessed. Participant knowledge was assessed based on basic knowledge, risk factors, and early screening knowledge. Correct and incorrect answers were assigned 1 and 0 point, respectively, with the highest total score being 10 points. The higher the score, the higher the knowledge awareness rate. Gynecological and breast disease-related behaviors included breast self-examination, clinical breast examination, and cervical cancer screening. Social support status was assessed using the validated Social Support Rating Scale. This instrument featured three dimensions (subjective support, objective support, and support utilization) and ten items, with an aggregate score that ranged from 7–56. Among these ten items, seven were answered on a four-point Likert scale, while the other items were answered by counting the number of sources of support. To determine the level of social support, the score index was classified into three; social support scores were considered “Poor” when they were below 25, “General” when they were between 25 and 37, “Relative” when they between 38 and 50, and “Satisfying” when they were 51 and above.

### Data analysis

#### Statistical analysis

The questionnaires were collected and double-entered using EpiData 3.1 software, and a database of the valid questionnaires was created. Descriptive analyses are presented as means and percentages. All statistical analyses were performed with custom-written or adapted scripts in the Python 3.10.6 and IBM SPSS 26.0 software.

#### Cluster analysis

**Fig. 1:**
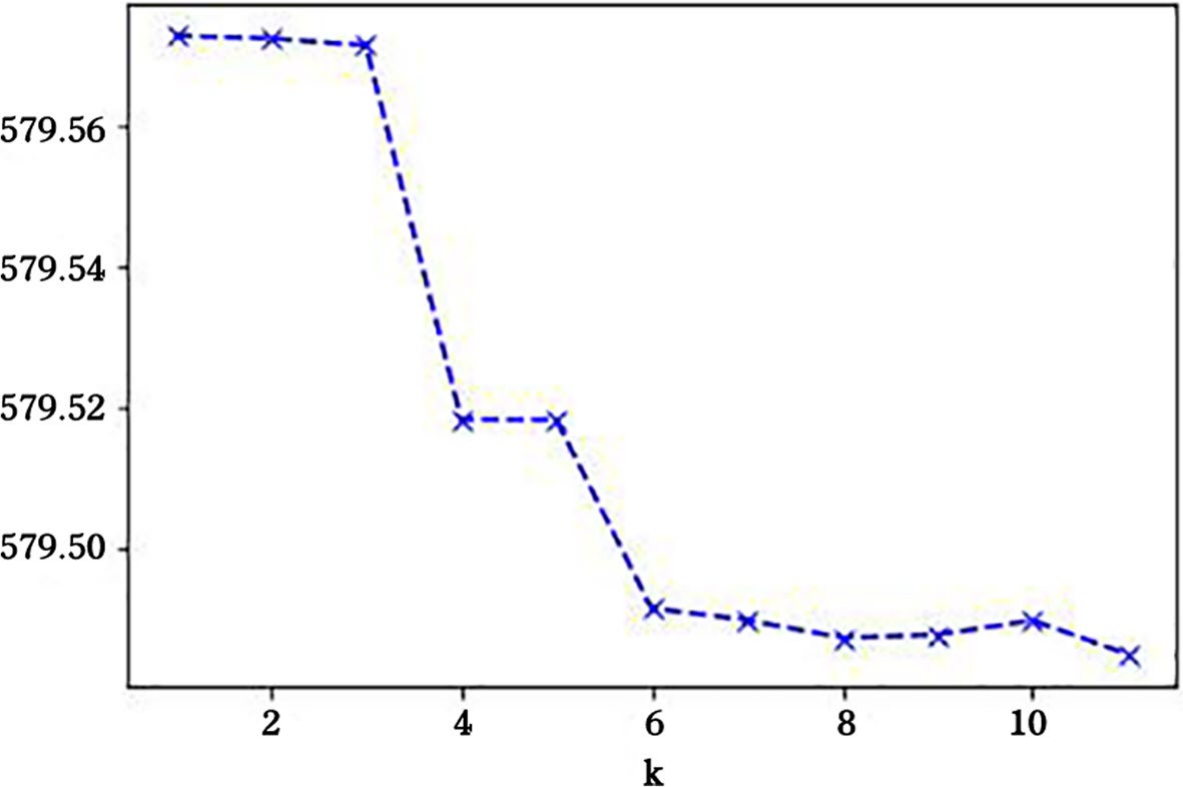
Diagram of k versus SSE. As the cluster number increases, the SSE (error sum of squares) trend changes. When the k value reaches the optimal cluster number, the SSE reduction amplitude suddenly becomes smaller and gradually tends flatten. The k value corresponding to this critical point is the optimal cluster number

The Python 3.10.6 sklearn toolkit was used to perform k-means unsupervised learning clustering analysis on five indicators in three dimensions, including illness, mental health status, and self-rated health status. Data were standardized and normalized before clustering to improve accuracy. As the number of clusters, k, increased and the sample was more finely divided, the degree of aggregation for each cluster gradually increased and the sum of squared errors (SSE) gradually became smaller. When k reached the true number of clusters, the return on the degree of aggregation obtained by increasing k again rapidly became smaller, and the decline in SSE plummeted and subsequently leveled off as the value of k continued to increase. Thus, the value of k corresponding to the inflection point in the plot of SSE versus k was the true number of clusters of the data. k-means clustering was performed after standardization of the five evaluation indicators for the 2,925 participants. The number of clusters selected for this study was determined to have a k-value of six (Fig. 1).

#### Correlations and regressions

To determine the possible relationship between sociodemographic characteristics, lifestyle behaviors, hygiene habits, social support, knowledge related to gynecological diseases, and different health patterns, we first performed univariate chi-square tests on all the variables. For the multivariable analyses, we used multi-factor logistic regression models to calculate odds ratios (OR) and 95% confidence intervals (CI) to investigate factors associated with health, the health status clustering results as the dependent variable, and the single significant term as the independent variable. *P *< 0·05 was considered statistically significant.

## Results

### Participant sociodemographic characteristics

Among the valid questionnaires, 2,925 questionnaires from participants aged 15–49 years were screened. The average participant age was 32.15 ± 8.61 years, with an approximately equal proportion of urban (58.9%) and rural (41.1%) participants. Most of the participants had a bachelor's degree or higher (62%) (Table 1).

**Table 1:** Basic sociodemographic profile of women of childbearing age in Shanxi Province

**Demographic characteristics**	**Number of people**(**N/%**)
**Age**	
< 27	949 (32.4)
27-	1208 (41.3)
≥ 39	768 (26.3)
**Household Registration**	
Urban	1724 (58.9)
Rural	1201 (41.1)
**Education level**	
Junior secondary school and below	193 (6.6)
Senior secondary school	282 (9.6)
Tertiary	637 (21.8)
Bachelor's degree	1343 (45.9)
Postgraduate and higher	470 (16.1)
**Marital Status**	
Unmarried	1113 (38.1)
Married	1757 (60.1)
Other	55 (1.9)
**Annual household income per capita**	
< 10,000	550 (18.8)
10,000-	403 (13.8)
20,000-	422 (14.4)
30,000-	537 (18.4)
50,000-	685 (23.4)
≥ 100,000	328 (11.2)
**Occupation**	
State institutions/institutions	299 (10.2)
Enterprise workers	652 (22.3)
Business services personnel	128 (4.4)
Health care workers	451 (15.4)
Educators	437 (14.9)
Transport staff	27 (0.9)
Self-employed	98 (3.4)
Students	491 (16.8)
People working in agriculture, fisheries and livestock	29 (1.0)
Temporary and unemployed workers	153 (5.2)
Other	160 (5.5)
**Type of medical insurance**	
Urban employees’ insurance medical	1545 (52.7)
Urban and rural residents' insurance medical	967 (33.0)
Publicly funded health care	79 (2.7)
Medical assistance	23 (0.8)
Commercial health insurance, purchased by the unit	169 (58)
Commercial health insurance, individual purchase	366 (12.5)
Major medical insurance for urban jobless residents	34 (1.2)
No insurance	96 (3.3)
Other	34 (1.2)

### Health pattern groups

**Fig. 2:**
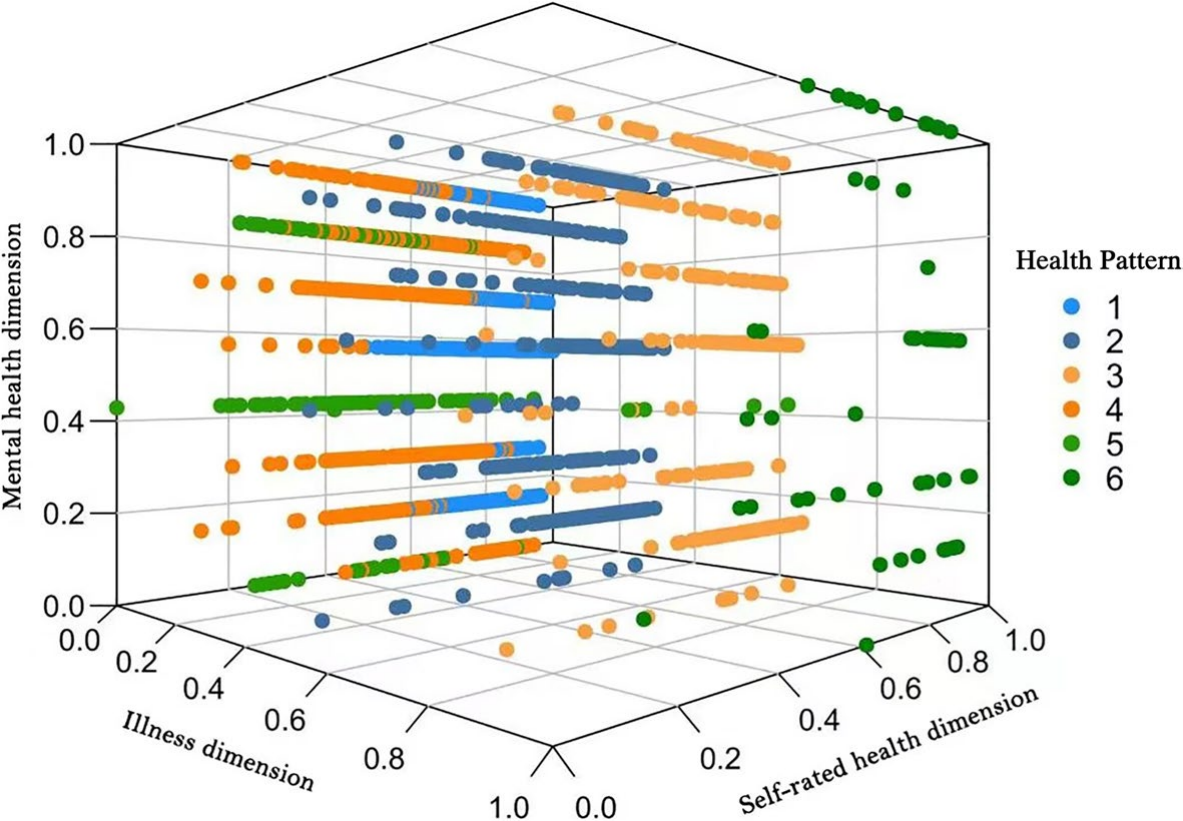
Clustered scatter plot. Visualization of mental health, illness, and self-rated health dimensions of individuals with different health patterns

The 2,925 participants were sorted into six clusters, and the centroid of each health mode is shown in Table 2. A total of 1,258 participants (43.0%) were classified into Health Pattern 1, signifying good health status in all five domains along three dimensions, including quality of life, mental health, and illness. A total of 499 (17.1%) participants were classified into Health Pattern 2, signifying a slightly lower quality of life and mental health status, and worse chronic disease status. A total of 288 (9.8%) participants were classified into Health Pattern 3, signifying a slightly lower quality of life and mental health status and worse health status at two weeks prior to the questionnaire. A total of 647 (22.1%) participants were classified into Health Pattern 4, signifying a much lower quality of life and mental health. Meanwhile, 166 (5.7%) participants were classified into Health Pattern 5, signifying the lowest level of quality of life and mental health status. Finally, 67 participants (2.3%) were classified into Health Pattern 6, signifying a slightly lower level of quality of life and mental health status and the worst disease status (Table 2). The scatter plots of the individual health patterns are shown in Fig. 2.

**Table 2:** Clustered mass centers for each health pattern

Health Pattern	Quality of life	Depression	Anxiety	Health status in a fortnight	Chronic disease prevalence
1	0.810	0.116	0.038	0.994	0.000
2	0.730	0.200	0.124	0.989	1.000
3	0.719	0.209	0.122	0.206	0.000
4	0.624	0.291	0.197	0.992	0.000
5	0.489	0.514	0.575	0.974	0.006
6	0.712	0.164	0.128	0.209	1.000

After standardization and normalization of all variables, all variable values are between 0 and 1. For QOL, values closer to 1 indicate better QOL. A depression score closer to 1 indicates more severe depression. An anxiety score closer to 1 indicates more severe anxiety. Scores closer to 1 for illness over two weeks indicate better health. A chronic disease prevalence value of 1 indicates a chronic disease

### Health status

The mean SF-12 scale score was 579.88 ± 107.97. In the distribution analysis of the number of people in each Health Pattern by age, education level, income level, and marital status, the distribution of health patterns in the different income groups was roughly the same as the distribution trend of the six types of health patterns in the overall survey population.

**Fig. 3:**
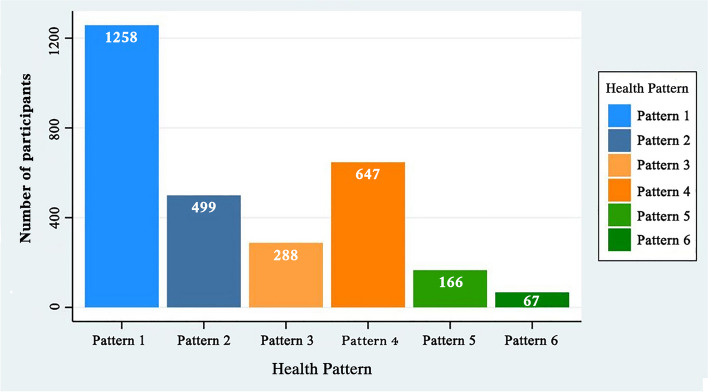
Participant distribution by health pattern. The number of participants in each health pattern

**Fig. 4:**
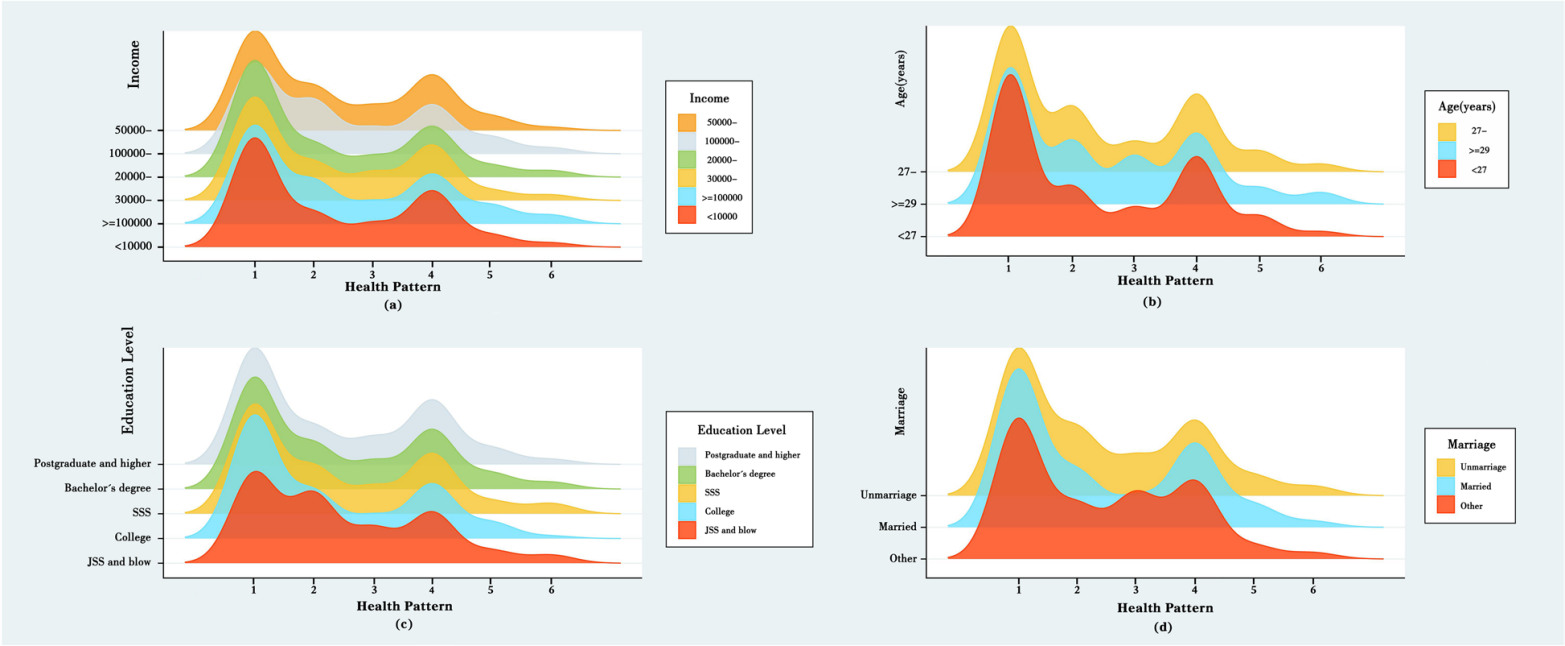
Health pattern distribution by age, income, culture, and marital status. **a** The number of participants across different (**a**) income groups, (**b**) age groups, (**c**) education level groups, and (**d**) marriage groups in six health patterns

The distribution map of the health patterns is shown in Fig. 3. Figure 4a to d illustrate the distribution of health patterns according to income, age, education level, and marriage, respectively. Figure 4c illustrates the distribution of the six health patterns among women of childbearing age categorized into different literacy level subgroups. Notably, there was a significant deviation in the distribution trend of the six health patterns among participants with junior high school education and below (red color block) compared with the overall survey population stratified by education level. Specifically, there was a substantial decrease in the number of participants in Health Pattern 1 and a significant increase in the number of participants categorized into Health Pattern 2. Women with junior high school education and below exhibited a lower proportion of individuals in the three-dimensional (3-D) health pattern and a higher percentage of individuals with disease conditions, particularly chronic diseases, in comparison to participants with higher educational levels. These findings suggest that individuals with lower educational attainment may have a diminished presence in the 3-D health pattern due to a higher prevalence of chronic diseases. In Fig. 4d, women with other marital statuses (divorced or widowed) demonstrated poorer representation in the 3-D health pattern and a higher proportion in Health Pattern 3, indicating that this subgroup represents a smaller portion of the 3-D health pattern population due to a higher prevalence of the two-week disease status.

### Correlations and regressions

Table 3 presents the final multivariate logistic regression data. Considering Health Pattern 1 (optimal health pattern) and Health Pattern 2 (poor chronic disease status), participants with education levels of senior high school (OR = 0.462, 95% CI: 0.283–0.753), tertiary (OR = 0.520, 95% CI: 0.312–0.865), bachelor’s degree (OR = 0.516, 95% CI: 0.329–0.810), and postgraduate degree and higher education (OR = 0.584, 95% CI: 0.380–0.896) showed better performance in the 3-D health pattern than that showed by those with education levels of junior high school and below. A lean meat diet was associated with a higher risk of poor health status than that observed with a balanced diet (OR = 1.455, 95% CI: 1.044–2.027). Compared with those who never had a gynecological examination, those who had regular gynecological examinations (OR = 1.842, 95% CI: 1.313–2.585), irregular gynecological examinations (OR = 1.469, 95% CI: 1.039–2.076), and gynecological examination only when physical abnormalities were found (OR = 1.532, 95% CI: 1.039–2.076) had better performance in the 3-D health pattern. Meanwhile, women with unhealthy family members were associated with a higher risk of poor health status than that observed in women with healthy family members (OR = 1.473, 95% CI: 1.185–1.831). Women with poor access to health care (OR = 1.452, 95% CI: 1.004–2.101) showed worse performance in the 3-D health pattern than that showed by women with better access to health care.

**Table 3:** Multivariate logistic regression analysis of correlates of health status of women of childbearing age

Variable	1 vs 2		1 vs 3		1 vs 4		1 vs 5		1 vs 6	
	*P*-value	OR (95% CI)	*P*-value	OR (95% CI)	*P*-value	OR (95% CI)	*P*-value	OR (95% CI)	*P*-value	OR (95% CI)
**Age**										
<27 years vs ≥39 years^R^	0.146	0.795 (0.583-1.083)	<0.001^*^	0.434 (0.302-0.625)	0.347	0.875 (0.663-1.155)	0.659	0.896 (0.551-1.458)	0.015^*^	0.403 (0.193-0.841)
27 years - vs ≥39 years^R^	0.740	1.047 (0.797-1.376)	<0.001^*^	0.551 (0.397-0.764)	0.722	1.048 (0.810-1.355)	0.605	1.127 (0.716-1.772)	0.116	0.623 (0.345-1.124)
**Literacy level**										
SSS vs JSS and below^R^	0.002^*^	0.462 (0.283-0.753)	0.459	0.799 (0.440-1.449)	0.808	0.942 (0.580-1.529)	0.828	0.911 (0.391-2.122)	0.455	0.652 (0.213-2.000)
College vs JSS and below^R^	0.012^*^	0.520 (0.312-0.865)	0.543	0.821 (0.436-1.548)	0.952	1.016 (0.608-1.699)	0.698	0.833 (0.332-2.094)	0.622	1.305 (0.452-3.769)
Bachelor's degree vs JSS and below^R^	0.004^*^	0.516 (0.329-0.810)	0.193	0.682 (0.384-1.213)	0.333	0.793 (0.495-1.269)	0.881	0.939 (0.416-2.124)	0.053	0.306 (0.092-1.017)
Postgraduate and higher vs JSS and below^R^	0.014^*^	0.584 (0.380-0.896)	0.757	0.919 (0.538-1.569)	0.784	0.939 (0.599-1.472)	0.914	0.957 (0.437-2.097)	0.964	1.022 (0.392-2.665)
**Diet**										
Vegan vs Balanced^R^	0.200	0.832 (0.627-1.103)	0.158	1.257 (0.915-1.725)	0.953	0.993 (0.779-1.265)	0.098	0.684 (0.436-1.073)	0.938	1.025 (0.548-1.90)
Meatier vs Balanced^R^	0.027^*^	1.455 (1.044-2.027)	0.010^*^	1.669 (1.128-2.470)	0.459	1.126 (0.823-1.540)	0.633	0.876 (0.509-1.509)	0.111	1.793 (0.875-3.677)
**Cooking Preferences**										
Heavy on oil and salt vs Moderate^R^	0.221	0.805 (0.570-1.139)	0.180	1.301 (0.886-1.910)	0.624	1.076 (0.802-1.445)	0.426	1.111 (0.745-2.005)	0.260	1.517 (1.079-3.135)
Light vs Moderate^R^	0.824	0.969 (0.731-1.283)	0.167	1.267 (0.906-1.772)	0.129	1.211 (0.946-1.551)	0.746	1.077 (0.687-1.688)	0.083	1.706 (0.932-3.123)
Sweet vs Moderate^R^	0.858	1.055 (0.584-1.906)	0.004^*^	2.311 (1.299-4.110)	0.261	1.336 (0.806-2.217)	0.021^*^	2.337 (1.137-4.799)	0.390	1.726 (0.498-5.986)
**Health status of family members**										
Poor vs Better^R^	<0.001^*^	1.473 (1.185-1.831)	0.767	0.959 (0.729-1.262)	0.948	0.993 (0.813-1.214)	0.171	0.779 (0.545-1.114)	0.084	1.561 (0.942-2.588)
**Hand washing**										
Poor vs Better^R^	0.743	0.934 (0.619-1.408)	0.011^*^	1.693 (1.126-2.546)	0.780	1.052 (0.737-1.502)	0.614	1.164 (0.645-2.099)	0.050^*^	2.047 (1.000 - 4.229)
**Gynaecological examination**										
Regularly vs Never ^R^	<0.001^*^	1.842 (1.313-2.585)	0.857	1.038 (0.693-1.553)	0.659	0.937 (0.702-1.251)	0.375	1.277 (0.759-2.147)	0.022^*^	2.716 (1.154 - 6.394)
Irregularly vs Never ^R^	0.029^*^	1.469 (1.039-2.076)	0.251	1.254 (0.852-1.845)	0.521	0.910 (0.683-1.213)	0.089	1.517 (0.939-2.452)	0.088	2.153 (0.892-5.198)
When physical abnormalities vs Never ^R^	0.016^*^	1.532 (1.082-2.170)	0.318	1.218 (0.827-1.794)	0.351	1.141 (0.865-1.506)	0.106	1.491 (0.919-2.421)	0.287	0.644 (0.658-4.112)
**Knowledge of cervical cancer**										
Unconversant vs Conversant^R^	<0.001^*^	1.556 (1.242-1.950)	0.610	0.930 (0.702-2.100)	0.946	0.993 (0.807-1.221)	0.733	0.940 (0.657-1.344)	0.938	1.021 (0.602-1.732)
**Breast cancer prevention attitudes**										
Positive vs Medium^R^	0.053	1.729 (0.994-3.009)	0.704	0.861 (0.398-1.861)	0.898	0.964 (0.548-1.695)	0.073	1.940 (0.941-4.001)	0.994	0.995 (0.225-4.394)
Negative vs Medium^R^	0.154	1.174 (0.942-1.465)	0.150	0.819 (0.624-1.075)	0.038^*^	1.235 (1.012-1.508)	0.686	0.930 (0.655-1.321)	0.918	0.974 (0.583-1.625)
**Reproductive health information**										
Poor vs Medium^R^	0.392	0.868 (0.628-1.200)	0.435	1.164 (0.795-1.704)	0.604	0.926 (0.694-1.237)	0.004^*^	1.974 (1.242-3.135)	0.455	0.737 (1.640-0.331)
Better vs Medium^R^	0.985	1.003 (0.776-1.296)	0.542	0.906 (0.659-1.245)	0.482	0.920 (0.730-1.160)	0.807	0.947 (0.613-1.463)	0.652	0.874 (0.488-1.567)
**Accessibility**										
Worse vs Better^R^	<0.048^*^	1.452 (1.004-2.101)	0.133	1.380 (0.907-2.100)	0.162	1.252 (0.914-1.717)	0.001^*^	2.178 (13.960 - 3.399)	0.423	0.612 (0.185-2.033)
**Social support**										
Poor vs Satisfying social support^R^	0.147	1.929 (0.793-4.692)	0.009^*^	4.536 (1.448-14.206)	<0.001^*^	4.577 (2.002 - 10.467)	0.001^*^	9.310 (2.380 - 36.423)	0.124	2.680 (0.782-9.650)
General vs Satisfying social support^R^	0.407	1.210 (0.771-1.901)	0.003^*^	3.082 (1.472-6.453)	<0.001^*^	3.167 (1.921-5.221)	0.002^*^	5.070 (1.776-14.477)	0.124	2.730 (0.759-9.820)
Relative vs Satisfying social support^R^	0.128	1.376 (0.912-2.077)	0.009^*^	2.599 (1.275-5.299)	0.001^*^	3.184 (1.349-3.538)	0.094	2.423 (0.859-6.837)	0.125	2.568 (0.770-8.560)

^*^For *p*-value less than 0.05

^R^ means Reference, which means it serves as a control group. In the analysis, the coefficients (parameter estimates) of the other groups are relative to the control group

A comparison between Health Pattern 1 (optimal health pattern) and Health Pattern 3 (poorer health in the last two weeks) populations showed that younger age (< 27 years: OR = 0.434, 95% CI: 0.302–0.625; 27–38 years: OR = 0.551, 95% CI: 0.397–0.764) was associated with higher health protection. A lean meat diet was associated with a higher risk of poor health status than that observed with a balanced diet (OR = 1.669, 95% CI: 1.128–2.470). A preference for sweeter cooking was associated with a higher risk of poor health status compared with that observed with moderate cooking preference (OR = 2.231, 95% CI: 0.299–4.110). Regarding social support, participants with poor social support (OR = 4.5363, 95% CI: 1.448–14.206), general support (OR = 3.082, 95% CI: 1.472–6.453), and relative support (OR = 2.599, 95% CI: 1.275–5.299) showed worse performance in the 3-D health pattern than that showed by those with satisfactory social support. A poor hand washing habit was associated with a higher risk of poor health status than that observed with a good hand washing habit (OR = 1.693, 95% CI: 1.126–2.546).

Comparison between Health Pattern 1 (optimal health pattern) and Health Pattern 4 (poor self-rated health) populations showed that a negative attitude toward breast cancer prevention was associated with a higher risk of poor health status than that observed with a positive attitude (OR = 1.235, 95% CI: 1.012–1.508). In addition, participants with poor social support (OR = 4.577, 95% CI: 2.002–10.467), general support (OR = 3.167, 95% CI: 1.921–5.221), and relative support (OR = 3.184, 95% CI: 1.349–3.538) showed worse performance in the 3-D health pattern than that showed by those with satisfactory social support.

Comparison between the Health Pattern 1 (optimal health pattern) and Health Pattern 5 (worst self-rated health) populations showed that a preference for sweeter cooking was associated with a higher risk of poor health status than that observed with a moderate cooking preference (OR = 2.337, 95% CI: 1.137–4.799). Participants with poor social support (OR = 9.310, 95% CI: 2.380–36.423), general support (OR = 5.070, 95% CI: 1.776–14.477), and relative support (OR = 1.974, 95% CI: 1.242–3.135) showed worse performance in the 3-D health pattern than that showed by those with satisfactory social support. Women with poor access to health care (OR = 2.178, 95% CI: 13.960–3.399) showed worse performance in the 3-D health pattern than that showed by those with better access to health care.

In comparing the Health Pattern 1 (optimal health pattern) and Health Pattern 6 (poor prevalence of both illness and chronic disease in the last two weeks) populations, being < 27 years old was associated with higher health protection (OR = 0.403, 95% CI: 0.193–0.841). Having regular gynecological examinations (OR = 2.716, 95% CI: 1.154–6.394) and poor hand washing habits (OR = 2.047, 95% CI: 1.000–4.229) were associated with a higher risk of poor health.

## Discussion

Women’s health encompasses multiple dimensions and is shaped by a wide range of factors, including physical, mental, social, and reproductive aspects. Using an integrated approach that considers all these facets is crucial for a comprehensive assessment of a woman’s health status. In contrast, relying on a singular approach that focuses solely on one aspect may neglect other significant health considerations. Through a comprehensive assessment, potential health risks and underlying conditions that could impact a woman’s reproductive health or future pregnancies can be identified. This approach enables the early detection of chronic diseases, genetic disorders, and mental health issues. Timely recognition of these risks allows for prompt intervention, management, and support, thereby optimizing health outcomes for women and potential children. Women’s health is influenced by various sociocultural, economic, and environmental factors. Comprehensive evaluations play a vital role in uncovering disparities in health outcomes and access to healthcare services among diverse groups of women. This understanding is pivotal in designing targeted interventions and formulating policies that address specific needs and work towards reducing health disparities.

This study observed that less than half of the women of childbearing age had optimal health patterns in Shanxi Province, indicating that women’s health awareness is increasing as China’s economic level and women’s social status are improving. Within the comprehensive evaluation system employed in this study, participants’ self-rated health, serving as a subjective self-assessment, offers insights into overall health status. Comparing the optimal health pattern (Health Pattern 1) with other patterns, Health Patterns 2, 3, and 6 showed slightly worse self-rated health, likely attributed to the presence of two weeks of prior illness and chronic disease. Health Patterns 4 and 5 exhibited poorer self-rated health, potentially due to inferior mental health. Furthermore, the impact of illness on self-rated health is considered less significant than the impact of mental health status. This discrepancy may arise from the subjectivity of self-assessed health, which is influenced by personal perceptions. Therefore, individuals with chronic illnesses may still rate their health positively if they perceive effective management or minimal impact on their daily lives. Conversely, individuals with depression and anxiety may rate their overall health lower, even in the absence of physical illness. Notably, physical and mental health are interconnected, where changes in one domain can influence the other. For instance, individuals with chronic physical illnesses may experience psychological distress or depression due to limitations or effects on daily life. Similarly, poor mental health can contribute to the development or exacerbation of physical health conditions.

We found that lifestyle behaviors compared with demographic characteristics had more influence on the health status of women of childbearing age and that lifestyle played a crucial role influencing health and disease. An unhealthy lifestyle is one of the top ten causes of death in the United States [23] and is a significant causal factor in the top ten diseases in China [24]. For example, eating a meat-heavy diet is a risk factor for poor health and is a long-term habit that may lead to unbalanced nutrient intake, negatively impacting an individual’s health status. Several experimental models and studies have shown that a shift to a more plant-based diet, with a lower consumption of red and processed meat and a higher consumption of fruits and vegetables, can reduce the risk of life-threatening diseases [25].

Regression analysis comparing Health Pattern 1 with Health Patterns 2 and 6 showed that regular gynecological check-ups were associated with a higher risk of poor health status. This is likely because gynecological examinations are often included as part of a health check-up. Therefore, these women are more likely to be diagnosed with chronic diseases because they undergo more frequent check-ups. Meanwhile, the chronic conditions of women who had never had a gynecological check-up were not detected. Therefore, the women who do not go for regular check-ups may have better self-reported health regarding chronic diseases due to the ignorance of their chronic disease status, rather than its absence. Although gynecological screenings can help screen asymptomatic women for gynecological conditions, such as ovarian and cervical cancer, no studies have directly assessed the effectiveness of pelvic examinations for improving health outcomes, such as quality of life, morbidity, or mortality [26]. Therefore, some guidelines do not recommend pelvic screening in asymptomatic, non-pregnant adult women [27, 28]. However, the American College of Obstetrics and Gynecology recommends annual pelvic examinations for all patients aged at least 21 years [29].

Among the 2,925 women of childbearing age surveyed in this study, 1,680 women reported washing their hands every time after using the toilet. Although we cannot rule out the tendency for people to change their behavior under observation or to overreport based on expectations [30], we believe that these women of childbearing age wash their hands more frequently after using the toilet. The results of the regression analysis comparing Health Pattern 1 with Health Patterns 3 and 6 showed that younger people and those with good hand washing habits had better health status regarding recent illnesses. This is consistent with the findings by Freeman et al. that suggest that hand washing after contact with excreta may have positive health benefits; however, hand washing is rarely practiced globally [31]. This suggests the need for better health education on hand washing hygiene, especially with the current COVID-19 pandemic [32], wherein hand washing has been associated with a reduction in disease incidence.

Social support refers to a person’s perception of the support they receive from others, such as a spouse, family member, friend, or healthcare professional. It is generally divided into instrumental support (help or assistance with tangible needs) and emotional support (beliefs of love and care, compassion, and understanding). The results of the regression analyses of the comparison between Health Pattern 1 and Health Patterns 3, 4, and 5 showed significant differences in the social support scales. The main differences between them were mainly in the three indicators requiring subjective judgment: self-rated health, depression, and anxiety. This result is consistent with that of previous research showing that social support can promote mental health and reduce the risk of psychopathology, especially depression [33].

Income had a small effect on the health status of women of childbearing age, possibly because China’s overall income improved with the improvement in its economy. Further, China has started focusing on improving the health and literacy of the population and reducing the burden of medical expenses by establishing a comprehensive health insurance system. Attitudes toward breast cancer prevention differed significantly between Health Pattern 1 and Health Pattern 4 populations, with negative mental health status being associated with negative attitudes toward preventive care. Attitudes toward breast cancer prevention reflect individual attitudes toward preventive health care. Positive attitudes toward preventive care encourage individuals to prioritize their health and be willing to make changes in their daily behavior to maintain health. Knowledge of cervical cancer and its reproductive health reflects the level of interest in health-related issues and health literacy. People with lower health literacy have a poorer quality of life, shorter life expectancy, and unhealthy lifestyles and are more likely to experience depression [21, 34]. Adequate health literacy increases an individual’s ability to access, evaluate, and use health-related information and make appropriate health choices. Low health literacy can lead to the inappropriate use of health resources.

The strength of this study is the use of cluster analyses for health evaluation while focusing on the health status of women of childbearing age, analyzing the health self-assessment, mental health, and recent and long-term illness dimensions. To the best of our knowledge, this study is the first to explore the health status of women of childbearing age and its influencing factors after the first year of negative population growth in Shanxi in 2021. The limitation of this study is the use of convenience sampling as the sampling method, as this non-probability sampling method may have resulted in an underrepresented sample.

## Conclusions

The study findings suggest that the comprehensive health status of women of childbearing age in Shanxi Province is generally good. However, there remains a large proportion of women with deficiencies in some dimensions, such as the self-rated health, illness, and mental health dimensions. Among the influencing factors affecting the comprehensive health status of women of childbearing age, lifestyle had the greatest impact on women’s health, which may suggest targets for the development of interventions to enhance the health status of childbearing-aged women worldwide. In a future follow-up study, we plan to conduct a remote health intervention, including health education on lifestyle and health-related knowledge, to observe the effect on the health status of women of childbearing age.

### Supplementary Information

**Additional file 1.** 

## Data Availability

The raw data supporting the results of this study are available from the corresponding author upon reasonable request.

## Abbreviations

3-D: Three-dimensional
CI: Confidence interval
COVID-19: Coronavirus disease
OR: Odds ratio
SF-12: Short-Form Health Survey 12
SSE: Sum of squared errors
