# Supplementary material for: Unsupervised clustering analysis of comprehensive health status and its influencing factors on women of childbearing age: a cross-sectional study from a province in central China
Source: BMC Public Health. 2023 Nov 9;23:2206. doi: 10.1186/s12889-023-17096-3 (PMC10634171; doi:10.1186/s12889-023-17096-3)
Supplement: Supplementary file 1 — Additional file 1. [file 12889_2023_17096_MOESM1_ESM.docx]

**Women's health-related behavior questionnaire**

Dear female friends,

We are the research team from Shanxi Medical University who are investigating “Women's health-related behaviors." The main purpose of this interview is to understand the status quo of women's health-related behaviors.The attention paid to women's health is not only conducive to improving their own health level, but also will affect the overall health level of the family and the society. In addition, we will carry out relevant publicity and education activities in the future. If you are interested, you can leave your mobile phone number at the end of the questionnaire. All the contents of this survey are used for statistical analysis only, and we will keep the information of you and your family members confidential.Thank you very much for your cooperation and time.

1. **Demographic and socioeconomic characteristics**

1. Your age:______ years old

2. Where you live: ______city______County / district______Township / town

3. Your registered residence : ①Rural ②Urban

4. Your highest level of education：

①Primary School and below ②Junior high School

③Senior high School or Technical Secondary School ④Tertiary

⑤Bachelor's degree ⑥Postgraduate and higher

5. Your occupation type:

①Staff members of state organs and public institutions ②Enterprise employees

③Business and services industry personnel ④Health personnel

⑤Teaching staff ⑥Transportation personnel

⑦Self-employed ⑧Student

⑨Engaged in agriculture, fishing, animal husbandry ⑩The retired person

⑪Temporary workers and unemployed people without formal employment

⑫ Other (please indicate)

6. Your current marital status:

①Unmarried ②Married ③Live apart

④Dissociaton ⑤Bereft of one's spouse ⑥Other

7. Your average annual household income（RMB）:

①＜10000 ②10,000- ③20,000

④30,000- ⑤50,000- ⑥≥100,000

8. Do you currently participate in the following medical insurance policy (with multiple options)?

①Medical insurance for urban workers

②Medical insurance for urban and rural residents (combined with urban residents and the new rural cooperative medical insurance)

③Socialized medicine

④Medical assistance

⑤Commercial medical insurance, the unit to purchase

⑥Commercial medical insurance, where individuals who buy a

⑦Serious illness medical insurance for unemployed urban residents

⑧The Long-Term Care Insurance

⑨No insurance is available to the

⑩Other medical insurance policy, please indicate

1. **2. Health status and health-related behaviors**

**(1) Health status**

1. You think your physical health condition is:

①Very poor ②Poor ③Fair ④Good ⑤Excellent
2. Did you feel unwell (with injury or chronic disease) within two weeks before the investigation?

①Consciously physical discomfort, medical units for medical treatment

②Consciously feel unwell, self-administered medication

③He felt unwell, did not go to medical treatment, did not take any medication, but took work, suspended or stayed in bed for more than 1 day due to physical discomfort

④No physical discomfort

3. Whether you had a doctor-diagnosed chronic disease within six months prior to the survey:

①Hypertension ②Diabetes mellitus ③Coronary disease

④Stomgastric or duodenal ulcer ⑤Acute and chronic gastritis

⑥Cervical spondylopathy ⑦Rheumatoid arthritis ⑧Carcinoma of the lungs

⑨Cancer of the liver ⑩Gastric cancer ⑪Colon, rectum, and anus cancer

⑫Mammary cancer ⑬Cervical carcinoma ⑭Fibroid

⑮Ovarian tumor ⑯Coleitis ⑰None

4. Your diet and nutrition collocation: ①Balanced ②Meatier ③Vegan

5. Breakfast Habits: ①Everyday ②Occasional ③Never

6. Whether the three meals are regular: ①yes ②No

7. Your cooking style is compared to the local average person:

①Heavy on salt ②Moderate ③Light ④Sweet ⑤Heavy on oil

8. What is the health status of the family members?

①Very poor ②Poor ③Normal ④Good ⑤Fine
9. Sickness situation of family members:

9.1 Relationship with me ( ); disease: ( )

9.2Relationship with me ( ); the disease: ( )

**(2) Lifestyle**

1. Do you smoke regularly?

2. Do you drink alcohol regularly?

①Never ②Rarely ③Sometimes ④Often ⑤everyday

3. How many drinks (250ml / cup) of water a day?

①1-3 cups ②4-5 cups ③6-8 cups ④8-10 cups ⑤More than 10 cups

4. You sleep on average every day_______hours.

5. Do you often take part in physical exercise?

①Never ②Rarely ③Sometimes ④Often ⑤everyday

6. You feel the pressure of your career (work) feeling now:

①Very high ②High ③General ④Low ⑤Very low

**(3) Health habits**

1. Do you wash your hands often after using the toilet?

①Never ②Rarely ③Sometimes ④Often ⑤everyday

2. Do you often take a bath?

①Everyday ②Once every two days ③Once every three days

④Once a week ⑤Depending on the weather

3. Do you share daily necessities with your family?

①Never ②Rarely ③Sometimes ④Often ⑤everyday

4. Do you often change your underwear?

①Everyday ②Once every two days ③Once every three days

④Once a week ⑤Depending on the weather

5. Do you often wash your private parts?

①Everyday ②Once every two days ③Once every three days

④Once a week ⑤Depending on the weather

6. Do you have regular gynecological examinations?

①Regularly ②Irregular ③Something wrong with your body ④Never

1. **3. Breast cancer knowledge, attitudes, and behavior questionnaires**

**(1) Knowledge of breast cancer and early screening**

1. My family does not have breast cancer, so I am not at risk of developing the disease. ①Yes ② No

2 Eating too much fatty foods increases women's risk of breast cancer.  ①Yes ② No

1. Obese people are prone to breast cancer. ①Yes ② No

4 Long-term intake of oestrogen increases the risk of developing breast cancer.

①Yes ② No

5 The early symptom of breast cancer is a lump in the breast. ①Yes ② No

1. wrinkled or sunken breast skin may be a symptom of breast cancer. ①Yes ② No
2. Water or bleeding may be an early sign of breast cancer. ①Yes ② No
3. Breast or axillary pain is a sign of breast cancer. ①Yes ② No
4. Breast Self-examination is to help you familiarize yourself with the appearance and touch of your breasts. ①Yes ② No
5. The purpose of breast self-examination is to detect breast disease in time.

①Yes ② No

1. Breast B-ultrasound examination (molybdenum target) is one of the methods for early detection of breast cancer. ①Yes ② No

12 An annual physical examination allows for the early detection of breast cancer.

①Yes ② No

13 Women should begin a breast self-examination at the age of 20. ①Yes ② No

14 Breast self-examination should be performed monthly. ①Yes ② No

15 Breast self-examination should be performed three to five days after the end of menstruation. ①Yes ② No

16 menopausal women or near menopausal women should undergo breast self-examination at the same time of each month. ①Yes ② No

**(2) Breast cancer prevention attitude**

1. Regular breast examinations can detect breast disease as soon as possible.

① Complete disagree ②Disagree ③Uncertainty ④Agree ⑤Complete agree

2. Early detection of breast cancer is important to save the lives of breast cancer patients.

① Complete disagree ②Disagree ③Uncertainty ④Agree ⑤Complete agree

3. Reasonable diet (eat more fruits, vegetables, soy products), a good lifestyle (quit smoking and alcohol, more exercise, etc.) can reduce the risk of breast cancer.

①Complete disagree ②Disagree ③Uncertainty ④Agree ⑤Complete agree

4. It is impossible to examine the breast by yourself to find an abnormal mass.

①Complete disagree ②Disagree ③Uncertainty ④Agree ⑤Complete agree

5. If you receive breast disease prevention guidance, will you follow the instructions to change your eating habits and lifestyle?

①Complete disagree ②Disagree ③Uncertainty ④Agree ⑤Complete agree

**(3) Early screening behavior of breast cancer**

1. Do you know how to perform a breast self-test? ①Understand ②Not understand

2. Have you ever had a breast self-examination? ①Yes  ②No

3. If you have, your breast self-examination is:

①Feel a touch

②Have time to do according to the specification, have no time on a simple touch

③Do by the standard method

4. Do you know that breast ultrasound and breast molybdenum target are examinations with different focus?

①Yes ②No

5. Have you ever had a clinical breast examination?

①Yes ②No- - - - -jump to (4) the accessibility of early screening for breast cancer

6. If so, about how long since your last clinical breast examination?

①<1 year ②1-2 years ③2-5 years (excluding 2 years) ④More than 5 years

**(4) accessibility to early screening for breast cancer**

1. Do you have time to attend regular breast examinations? ①Yes ②No

2. Whether the transportation is convenient for you to go to the hospital? ①Yes ②No

3. The cost of ordinary breast examination is 80 yuan / time. Do your economic conditions support you to check once a year? ①Yes ②No

1. **4. Cervical cancer knowledge, attitude, and behavior questionnaires**

**(1) Relevant knowledge of cervical cancer**

1. What do you think are the early symptoms of cervical cancer (multiple choice)?

①Contact bleeding ②Menostaxis ③Abnormal vaginal discharge

④Frequency and urgency ⑤Anemia ⑥Unknown

2. In your opinion, who is prone to cervical cancer (multiple choice)?

①Population of all ages ②Married women aged 40 – 60 years ③No sexual debut yet

④Multiple pregnancies ⑤With multiple sexual partners ⑥Smoker

⑦cervical cancer family history ⑧Poor personal hygiene habits ⑨Unknown

3. What do you think is the most important cause of cervical cancer (multiple choice)?

①inherent cause ②sexually transmitted disease ③HPV virus infections

④Too many sexual partners ⑤bacillosis ⑥Poor hygiene habits

⑦Lifestyle factors ⑧Other

4. What age group do you think women should be screened for cervical cancer?

①20 married woman ②30 married woman ③Premenopausal women

④Postmenopausal women ⑤Other, ____please indicate ⑥hear nothing of

5. How do you think HPV is mainly transmitted through?(Note: HPV refers to human papillomavirus, which is A genus of papillvacuolar virus A belonging to the viridae family. It is A spherical DNA virus and can cause squamous epithelial proliferation of human skin mucosa.）

①Air-borne infection ②Gastrointestinal transmission

③Sexual transmission ④All of the above

6. Men are not infected with HPV.

①Yes ②No ③Indeterminacy

7. What you think is that HPV infection may cause (multiple choice):

①Cervical cancer ②Carcinoma of penis ③Colorectal cancer

④Pointed condyloma ⑤Other, please indicate ⑥Unknown

8. What do you think is the most effective way to prevent HPV infection?

①Regular use of condoms ②Vaccination against HPV ③Start having sex too early

④Avoid multiple sexual partners ⑤Other, please indicate

9. What do you think is the best time for HPV vaccination?

①Before first sex ②After first sex ③Other, please indicate ④Unknown

10. What do you think are the prevention methods for cervical cancer (multiple choice)?

①Change bad habits ②Reasonable diet ③A positive attitude

④fitting exercise ⑤HPV vaccination ⑥regular cervical cancer screening tests

⑦Other, please indicate ⑧Unknown

**(2) attitudes towards cervical cancer screening**

1. Do you think that HPV is highly susceptible to infection?

①Yes ②No ③Unknown

2. Are you worried that you may have an HPV infection?

①Yes ②No ③Unknown

3. Do you think the consequences of HPV infection are serious?

①Yes ②No ③Unknown

**(3) Behavior: cervical cancer screening status**

1. Have you ever been screened for cervical cancer before? ①Yes ②No

2. Are you worried about your risk of cervical cancer? ①Yes ②No ③Unknown

3. Are you willing to undergo cervical cancer screening in the future? ①Yes ②No

1. **reproductive health**

**Which of the following statements is correct (on the option):**

1. Menstrual cycle refers to the first day of each menstruation to the day before the next menstruation, which is generally 28 days

①Yes ②No ③Unknown

2. Menstruation early or delayed within 7 days belong to normal

①Yes ②No ③Unknown

3. Ovulation period is the next menstruation before the 14th day about

①Yes ②No ③Unknown

4. Easy pregnancy is the 12-18 days of the menstrual cycle

①Yes ②No ③Unknown

5. The optimal time for emergency contraception is within 72h

①Yes ②No ③Unknown

6. The best time for medical abortion is within 49 days of pregnancy

①Yes ②No ③Unknown

7. The best time to have an abortion is within 10 weeks of pregnancy, the earlier the better

①Yes ②No ③Unknown

8. Occasional sexual intercourse is not pregnant

①Yes ②No ③Unknown

9. The safest contraceptive against STD is condom

①Yes ②No ③Unknown

10. Premature sexual behavior can increase the risk of cervical cancer

①Yes ②No ③Unknown

1. **Social Support Rating Scale (SSRS)**

1. How many close friends can you have to get support and help (choose only one item)?

A. Nobody B. 1-2 C. 3-5 D. more than 6

2. In the past year (only one)?

A. Stay away from your family, and live alone in a room

B. Residence often changes, and most of the time he lives with strangers

C. Living with your classmates, colleagues or friends

D. Living with your family members

3. You and your neighbors (choose only one item)?

A . Never care about each other, just nod to friends

B. Encounter difficulties may be a little bit concerned

C . Some neighbors are very concerned about you

D. Most neighbors care about you

4. You and your colleagues (choose only one item)?

A . Never care about each other, just nod to friends

B. Encounter difficulties may be a little bit concerned

C . Some of my colleagues are very concerned about you.

D. Most of my colleagues care about you

5. Support and care from family members (mark "√") on the appropriate options

A. Husband and wife (lover) ① no ② rarely ③ general ④ full support

B. Parents ① no ② minimal ③ general ④ full support

C. Children ① no ② very little ③ general ④ full support

D. Siblings ① no ② minimal ③ general ④ full support

E. Other members (sister-in-law, etc.) ① no ② very little ③ general ④ full support

6. In the past, did you get financial support and help with practical problems?

(1) Without any source

(2) From the following sources (multiple choice)

A.Spouse B. Other Family Members C.friend D.Relative E.colleague

F.work unit G. An official or semi-official organization such as Party union

H. Non-governmental organizations such as religious and social organizations

I. Other (please listed)

7. Are the source of comfort and care you received when you encountered difficulties in the past?

(1) Without any source

(2) From the following sources (multiple choice)

A.Spouse B. Other Family Members C.friend D.Relative E.colleague

F.work unit G. An official or semi-official organization such as Party union

H. Non-governmental organizations such as religious and social organizations

I. Other (please listed)

8. How do you encounter trouble (choose only one)?

A. Never tell anyone B. Only recount to the close relationship of 1-2 people

C. If friends ask, you will say D. Proactively recount troubles to gain support and understanding

9. How do you seek help when you are in trouble (choose only one option)?

A . Only rely on oneself, do not accept the help of others

B. Rarely ask for help C. Sometimes I request help

D. When there are difficulties, I often ask family, relatives and organizations for help

10. For organizations (such as party and league organizations, religious organizations, trade unions, student unions, etc.), you (only one)

A.Never participate B.Occasionally participate

C.Frequent participate D.Actively participate

1. **The SF-12 scale**

1. In general, you think your current health status is:

①Excellent ②very good ③good ④Fair ⑤Poor

**The following two questions are about activities you might do during a typical day. Does YOUR HEALTH NOW LIMIT YOU in these activities? If so, how much?**

1. MODERATE ACTIVITIES, such as moving atable, pushing a vacuum cleaner, bowling, or playing Tai Chi:

①Yes, limited a lot ②Yes, limited a little ③No, not limited at all

3. Climbing SEVERAL flights of stairs:

①Yes, limited a lot ②Yes, limited a little ③No, not limited at all

**During the PAST 4 WEEKS have you had any of the following problems with your work or other regular activities AS A RESULT OF YOUR PHYSICAL HEALTH?**

4. ACCOMPLISHED LESS than you would like: ①Yes ②No

5. Were limited in the KIND of work or other activities: ①Yes ②No

**During the PAST 4 WEEKS, were you limited in the kind of work you do or other regular activities AS A RESULT OF ANY EMOTIONAL PROBLEMS (such as feeling depressed or anxious)?**

6. ACCOMPLISHED LESS than you would like: ①Yes ②No

7. Didn’t do work or other activities as CAREFULLY as usual: ①Yes ②No

8. During the PAST 4 WEEKS, how much did PAIN interfere with your normal work (including both work outside the home and housework)?

①Not At All ②A Little Bit ③Moderately

④Quite A Bit ⑤Extremely ⑥Not applicable

9. How much time have you felt calm and peaceful in the past four weeks?

①All of the Time ②Most of the Time ③A Good Bit of the Time

④Some of the Time ⑤A Little of the Time ⑥None of the Time

10. How much time have you felt energetic in the past four weeks?

①All of the Time ②Most of the Time ③A Good Bit of the Time

④Some of the Time ⑤A Little of the Time ⑥None of the Time

11. How much time have you felt downhearted and blue in the past four weeks?

①All of the Time ②Most of the Time ③A Good Bit of the Time

④Some of the Time ⑤A Little of the Time ⑥None of the Time

12. During the PAST 4 WEEKS, how much of the time has your PHYSICAL HEALTH OR EMOTIONAL PROBLEMS interfered with your social activities (like visiting with friends, relatives, etc.)

①All of the Time ②Most of the Time ③A Good Bit of the Time

④Some of the Time ⑤A Little of the Time ⑥None of the Time

1. **Self—Rating Depression Scale**

Please type on the corresponding form according to your situation in the last week.

| Place check mark "√" in correct column | <1 Day | 1-2 Days | 3-4 Days | 5-7 Days |
| --- | --- | --- | --- | --- |
| 1. I worry about some little things |  |  |  |  |
| 2. I find it hard to concentrate on doing things |  |  |  |  |
| 3.I feel down-hearted and blue |  |  |  |  |
| 4. I think it's very hard to do anything |  |  |  |  |
| 5. I have great hope for the future |  |  |  |  |
| 6. I feel afraid |  |  |  |  |
| 7. I don't sleep very well |  |  |  |  |
| 8. I'm happy |  |  |  |  |
| 9. I feel lonely |  |  |  |  |
| 10. I don't think I can go on with my life |  |  |  |  |

1. **Simple Self-assessment Scale of Anxiety Status (GAD-7)**

Please type on the corresponding form according to your situation in the last two weeks.

|  | Not at all | Several days | More than half the days | Nearly everyday |
| --- | --- | --- | --- | --- |
| 1. Feeling nervous, anxious or on edge |  |  |  |  |
| 2. Not being able to stop or control worrying |  |  |  |  |
| 3. Worrying too much about different things |  |  |  |  |
| 4. Trouble relaxing |  |  |  |  |
| 5. Being so restless that it is hard to sit still |  |  |  |  |
| 6. Becoming easily annoyed or irritated |  |  |  |  |
| 7. Feeling afraid as if something awful might happen |  |  |  |  |

**Your mobile phone number:**
